# Bayesian Mass Spectra Peak Alignment from Mass Charge Ratios

**Published:** 2008-04-11

**Authors:** Junfeng Liu, Weichuan Yu, Baolin Wu, Hongyu Zhao

**Affiliations:** 1 Department of Statistics, West Virginia University, Morgantown, WV 26506, U.S.A; 2 Departmentof Electronic and Computer Engineering, Hong Kong University of Science and Technology, Clear Water Bay, Sai Kung Kowloon, Hong Kong; 3 Division of Biostatistics, University of Minnesota, Minneapolis, MN 55455, U.S.A; 4 Department of Epidemiology and Public Health, Yale University School of Medicine, New Haven, CT 06520-8034, U.S.A

**Keywords:** Mass spectrometry, peak alignment, random grafting-pruning Markov chain Monte Carlo (RGP CMC), reversible jump Markov chain Monte Carlo (RJMCMC), sample classification, symmetric transition

## Abstract

Proteomics studies based on mass spectrometry (MS) are gaining popular applications in biomedical research for protein identification/quantification and biomarker discovery, especially for potential early diagnosis and prognosis of severe disease before the occurrence of symptoms. However, MS data collected using current technologies are very noisy and appropriate data preprocessing is critical for successful applications of MS-based approaches. Among various data preprocessing steps, peak alignment from multiple spectra based on detected peak sample locations presents special statistical challenges when effective experimental calibration is not feasible due to relatively large peak location variation. To avoid intensive tuning parameter optimization, we propose a simple novel Bayesian algorithm “random grafting-pruning Markov chain Monte Carlo (RGPMCMC)” that can be applied to global MS peak alignment and to follow certain model-based sample classification criterion for using aligned peaks to classify spectrum samples. The usefulness of our approach is demonstrated through simulation study by making extensive comparison with other algorithms in the literature. Its application to an ovarian cancer MALDI-MS data set achieves a smaller 10-fold cross validation error rate than other current large scale methodologies.

## 1. Introduction

Genomics and proteomics technologies offer much promise in our understanding of fundamental biological processes by allowing us to simultaneously monitor the expression levels of tens of thousands of genes and proteins. Since proteins are the basic functioning units in the cells, there is a great interest to characterize individual molecular profiles based on proteomics for reliable biomarker discovery and effective disease diagnosis/prognosis/treatment. In proteomics research, mass spectrometry (MS) is the most widely used instrument to allow for the mass measurement of molecules, where a mass spectrometer determines chemical compounds’ molecular weight by ionizing, separating, and measuring molecular ions according to their mass-to-charge ratio (m/z: unit Da) and a mass spectrum is the standard data output for analysis and interpretation (Link et al. 1999), where the x-axis represents m/z value (Da) and the y-axis represents intensity (enrichment of particles with certain m/z). Recently, Yu et al. (2005) reviewed current approaches on the extraction of the most relevant information from the raw mass spectra to identify disease biomarkers. A standard MS data analysis usually involves background noise removal, smoothing, intensity normalization, peak identification and alignment, and biomarker identification. The false positive and false negative peaks may exist in taking local maxima as peaks (Coombes et al. 2003) and peak location variation may be due to differences in sample preparation, chemical noise, co-crystallization, deposition of the matrix-sample onto the target, and laser position on the target among others. Several statistical methods have been proposed to reduce the background noise (Coombes et al. 2003; Satten et al. 2004; Coombes et al. 2005a and Randolph and Yasui, 2006). Morris et al. (2005) applied translation-invariant wavelet transformations to the raw spectra and performed peak detection using the mean spectrum derived from a group of spectra, where they assumed calibration can be done in advance experimentally or by interpolation to make common peaks stay closely together, and only false negatives are possible. However, Coombes et al. (2005b) pointed out that, peak location variation, caused by a spread of initial particle velocities at the starting end of mass spectrometer tube, makes calibration more difficult. Compared to background noise removal, peak identification and alignment is more challenging and critical by providing the links of underlying peptides across all spectra. However, existing algorithms for peak alignment are mostly ad hoc and based on heuristic arguments (Nielsen et al. 1998; Johnson et al. 2003; Torgrip et al. 2003; Eilers, 2004; Tibshirani et al. 2004 and Randolph and Yasui 2006), where some parameters need to be optimized empirically and/or subjectively. In this paper, we work on the detected peak samples (possibly with associated intensities) coming from certain protocol. In view of hundreds of features (peaks) in each spectrum, due to complex chemical and physical mechanisms undergoing the mass spectrometry, throughout this paper, we assume that substantial individual peak location variation is existent, say up to one half of the interval between neighboring peaks, thus calibration may be in lack of power. Moreover, we consider statistically false positives and negatives to make our model more accountable. Overall, we will assume that each set of potential peak samples corresponding to the true peak follows an individual composite distribution regulated by true peak location, peak sample location variation, false positive and false negative rates. An effective simple Bayesian MCMC algorithm is proposed to do peak alignment (biomarker identification) and downstream sample classification, where all individual sets of parameters for each true potential peak are able to be estimated in a universal modeling framework without intensive tuning parameter optimization. Our algorithm is much computationally simpler than other available MCMC algorithms which could be applied to MS alignment while retaining competing performance.

The rest of this article is organized as follows: Section 2 introduces Bayesian dimension matching problem and proposes our simplified approach; Section 3 summarizes simulation results to demonstrate the efficiency and reliability of our algorithm; Section 4 illustrates the applications of our approach to a real MS data set; Section 5 considers joint analysis of peak alignment and sample classification; Section 6 concludes with discussions; and some technical details are given in the appendix.

## 2. Methods

### 2.1. Dimension-matching statistical model

We now develop a simple novel MCMC algorithm, random grafting-pruning Markov chain Monte Carlo (RGPMCMC) which can be applied to MS peak alignment from mass charge ratio information. Within Bayesian framework, we are often interested in the posterior distribution of the dimension-varying parameter θ The prior distribution π(θ)is represented as ∑*_K_*_∈_*_N_* π(θ*_K_*|*K*)π(*K*), where N is positive integer set and π(θ*_K_* |*K*) is the individual prior distribution within the *K*-dimensional space and π(*K*)is the mixture probability for dimension *K*. Since π(*K*, θ*_K_*) = π(*K*)π(θ *_K_*|K), the posterior distribution of (*K*, θ*_K_*)

(1) $$\pi (K,\theta_{K}|X)=\frac{f(X|\theta_{K})\pi (\theta_{K})\pi (K)}{\sum_{KɛK}\left(\int_{\Theta_{K}}f(X|\theta_{K})\pi (\theta_{K})d\theta_{K})\pi (K\right)},$$

where the denominator is the normalization constant not needed for posterior sampling. Change point model usually involves dimension-matching in two typical cases: one single ordered series where change points are taken as those separating successive discrete points, and multiple ordered series where change points correspond to physical locations in the continuous space. For the discrete case, the partitioning and wrapping up the segments leads to an exponentially increasing computational cost as the model space grows (Denison et al. 2002), and frequentist’s approaches only either work on very few change points or special algorithms (Guan, 2004; Olshen and Venkatraman, 2004). Recently, Fearnhead (2005) proposed an exact non-MCMC sampler by recursive partition, and Loschi, Cruz and Arellano-Valle (2005) introduced the product partition model based Bayesian algorithm which stems from Yao (1984) and Barry and Hartigan (1992 and Barry and Hartigan (1993). For the continuous case, the readers are referred to sampling-based algorithms: the reversible jump MCMC (RJMCMC) by Green (1995), the birth-and-death process MCMC (BDMCMC) and continuous time process MCMC (CTMCMC) by Stephens (2000a, 2000b), Bayesian cluster detection in maps (Knorr-Held and Raßer, 2000) and others. Cappé, Robert and Rydén (2003) showed that, the acceptance probability of the usual MCMC methods is replaced by differential holding times in BDMCMC, and RJMCMC converges to a limiting continuous time birth-and-death process on an appropriate rescaling of time. They also demonstrated that, RJMCMC and CTMCMC have similar computational performance while the latter demands expensive death rate computation. Obviously, discretization is neither always suitable nor efficient for sophisticated change point identification in the continuous space. The present work introduces a simple MCMC algorithm in the context of multiple MS peak alignment by considering all uncertainties including peak number, peak locations, peak sample location variations, false negative and false positive rates. Neither the error-prone Jacobian terms in RJMCMC, the intensive death rate calculation in CTMCMC, nor the computationally expensive recursive partition in other algorithms is needed by our method.

**Figure 1:**
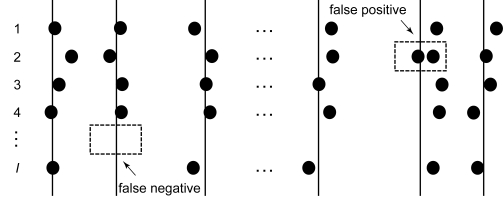
Mass Spectrum Biomarker Model. (The left column is spectrum index, the vertical lines are putative true peaks, the horizontal circle lines are peak samples of each spectrum. The lower left dash rectangle represents a false negative case and the upper right dash rectangle represents a false positive case.)

Before starting with our statistical model, we assume local maxima (discrete locations) have been detected as peak samples for each raw mass spectrum. In MS peak alignment, peak sample location variation may linearly depend on m/z magnitude (Yasui et al. 2003), and log-transformation of m/z achieves peak sample location variation homogeneity, this observation will justify the identical prior specification for peak sample location variations across true peaks (details later). For notational simplicity, we use m/z instead of log (m/z) in the following discussions and assume that the m/z domain is [(*m*/*z*)*_min_*, (*m*/*z*)*_max_*]. The underlying *K*-dimensional true peak location vector is *S̃_K_* = (*s*_1_, *s*_2_, …, *s_K_*), where the peak locations are generally separated by at least a distance threshold, say no less than *d*. The data to be analyzed from multiple spectra are the detected peak sample locations *y_ij_* (*j* = 1, …, *n_i_*, *i* = 1, …, *I*), where *i* is the index for spectra, *j* is the peak sample index within each spectrum (with increasing m/z), and *n_i_* is the number of detected peak samples in the *i*-th spectrum. The peak samples are assumed to be normally distributed around their true peak *s_k_* with standard deviation σ*_k_* (*k* = 1, …, *K*) for locations. For the putative true peak set *S̃_K_* = (*s*_1_, *s*_2_, …, *s_K_*), each detected peak sample *j* in *i*-th spectrum is assigned to its nearest putative true peak among *S̃_K_*, say *n_y_* (*i, j*) (with possible multiple assignments to the same putative peak from a given spectrum). For certain true peak *k*, (1) when there is no peak sample assignment to it from a given spectrum, we consider it as a false negative case for this spectrum with probability *fn_k_*; (2) when there are multiple peak sample assignments to it from a given spectrum, we consider it as a false positive case for this spectrum with probability *fp_k_*; (3) otherwise we consider it as a non-false positive or negative case for this spectrum with probability 1 − *fn_k_* − *fp_k_*. Our prior hypothesis is that, each true peak shows these three types of cases proportionally under the homogeneity assumption for the spectra and identical peak sample detection protocol for each local m/z region, say for each true peak, on average 90% spectra will contribute single peak sample, 5% will contribute multiple peak samples and 5% will contribute no peak sample to the putative true peak. For these three cases, we assume an independent trinomial distribution Tri(*I; fn_k_*, *fp_k_*, 1 − *fn_k_* − *fp_k_*) for each true peak *k* in the context of numbers of grabbed peak samples from *I* spectra. Now we restate the following notations for model set-up: ***Y*** stands for the peak sample locations for all *I* spectra, which need not be a *I*-row matrix since the numbers of peak samples are not necessarily equal because of false negatives and/or false positives; *S̃_K_* s a *K*-dimensional vector of putative true peak locations (m/z’s); σ̃*_K_* is a *K*-dimensional vector of peak sample location variations at putative true peaks; *f̃n_K_* and *f̃p_K_* are *K*-dimensional vectors of false negative and false positive rates at putative true peaks; *n_y_* (*i, j*) is putative true peak assignment to peak sample *j* in the *i*-th spectrum; *n_fn, k_* is the number of spectra without peak samples assigned to putative true peak *k; n_fp, k_* is the number of spectra with multiple peak samples assigned to putative true peak *k*, and $n_{\bar{fnp,\;}k}$ is the number of spectra with single peak sample assigned to putative true peak *k* (Figure 1). The likelihood for the observed peak samples across all spectra is

(2) $$\begin{array}{l} f(Y|K,{\tilde{S}}_{K},{\tilde{\sigma }}_{K},\tilde{f}n_{K},\tilde{f}p_{K})\\ =\prod_{i=1}^{I}\prod_{1\leq j\leq n_{i}}\varphi (y_{ij}|S_{n_{y}(i,j)},\sigma_{n_{y}(i,j)})\\ \times \prod_{k=1}^{K}{fn}_{k}^{n_{fn,\;k}}{fp}_{k}^{n_{fp,\;k}}{\left(1-{fn}_{k}-{fp}_{k}\right)}^{n_{\bar{nfnp,\;k}}}. \end{array}$$

We now describe the motivation for such a likelihood function which is crucial for Bayesian inference. The matching by minimum distance (*S_n_y_(i, j)_*) is to capture important clustering information. We believe that, this objective construction is reasonable compared to some alternatives, e.g. non-model based clustering algorithms, or piecewise binning method where each bin is assumed to hold exactly those peak samples for the individual putative true peak. The other part incorporating false positives and false negatives is mainly a technical statistical consideration, since it is very hard to accurately claim which peak sample is a true false positive or false negative. To sum up, in spite of substantial measurement errors underlying high-throughput mass spectra, we pursue a reasonable eclectic theme to tackle biomarker profile estimation. For notational convenience, “peak” represents putative true peak for the biomarker profile hereafter, other than detected peak sample from each spectrum. On the prior part, we assume that *K* follows a truncated Poisson or discrete uniform distribution on {*K_min_*, …, *K_max_*}. As Green (1995) suggested, the peak locations are taken as even-numbered order statistics from 2*K* + 1 points uniformly distributed on an *L*-length interval [(*m*/*z*)*_min_*, (*m*/*z*)*_max_*] (for convenience, *s*_0_ = (*m*/*z*)*_min_*, *s_K_*_+1_ = (*m*/*z*)*_max_*) to avoid too many short steps, which has density Π*_k_*_=1_*^K^*^+1^ (*s_k_* − *s_K_*_−1_)/*L*^2^*^K^*^+1^ as suggested by Green (1995). To make use of conjugate prior, π(σ^2^) is taken as Inverse-Gamma (ν, η) density (σ^2^)^−(ν+1)^ *e*^−1/(σ^2^η)^ η^−ν^/Γ(ν); the joint prior distribution for (*fn, fp,*1 − *fn* − *fp*) is a 3-dimensional Dirichlet distribution with density *D* (*fn*, *fp*, 1 − *fn* − *fp*|α_1_ α_2_, α_3_) = (*fn*)^α_1_−1^ (*fp*)^α_2_−1^ (1 −*fn* − *fp*)^α_3_−1^ Γ(α_1_)Γ(α_2_) Γ(α_3_)/Γ(α_1_ + α_2_ + α_3_). (The posterior distribution is

(3) $$\begin{array}{l} f(K,{\tilde{S}}_{K},{\tilde{\sigma }}_{K},\tilde{f}n_{K},\tilde{f}p_{K}|Y)\\ =\prod_{i=1}^{I}\prod_{1\leq j\leq n_{i}}\varphi (y_{ij};S_{n_{y}(i,j)},\sigma_{n_{y}(i,j)})\;\;\;(\text{Normal Dist}.)\\ \times \;\prod_{k=1}^{K}{fn}_{k}^{n_{fn,\;k}}{fp}_{k}^{n_{fp,\;k}}{\left(1-{fn}_{k}-{fp}_{k}\right)}^{n_{\bar{fnp,\;k}}}\;\;\;(\text{Trinomial Dist}.)\\ \times \;\pi (K,S_{K})\times \prod_{k=1}^{K}\left[D({fn}_{k},{fp}_{k},1-{fn}_{k}-{fp}_{k}|\alpha_{1},\alpha_{2},\alpha_{3}\right)\\ \times \;\prod_{k=1}^{K}IG(\sigma_{k}^{2}|\nu ,\eta )]\;\;\;(\text{Prior Dist}.) \end{array}$$

### 2.2. Random Grafting-pruning Markov chain Monte Carlo (RGPMCMC)

Motivated by Green (1995), our algorithm is based on a redesigned universal “naively informative” parameter proposal involving peaks and the other parameters concurrently without the need for Jacobian terms. We also propose four move types (+, −, *H, S*), where “+” means peak birth proposal, “−” means peak death proposal, “*H*” means parameter (σ^2^, *fn* and *fp*) proposal excluding peaks and “*S*” means peak location mutation with peak number unchanged. We specify (+, −, *H, S*) probabilities as (π(+),π(−), π(*H*), π(*S*)).

#### Parameter sampling process

**Figure 2:**
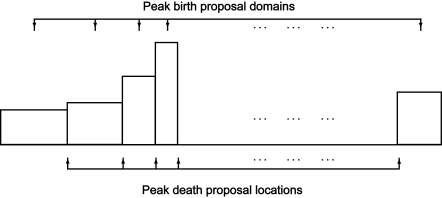
Symmetric Transition for Peak Birth/death Proposal. (Each rectangle represents a uniform peak birth proposal domain, each internal vertical boundary represents a possible peak death proposal location.)

1. First we choose one of these four move types based on move type probabilities (π(+),π(−), π(*H*), π(*S*)), where π(+) = π(−).
2. For the “+” move type, we describe the parameter proposal process: If *K_old_* = *K_max_*, we go to 1) since the upper threshold is reached; if *K_old_* < *K_max_*, we randomly sample one of the *K_old_* + 1 intervals formed by current *K_old_* peaks, say (*s_j_*, *s_j_*_+1_), with equal probability 1/(*K_old_* + 1). We may assign *j* + 1 to this new peak index * and the following indexes increase by one accordingly. Within this sampled interval, we propose (s_*_, σ_*_^2^, fn_*_ and fp_*_) for peak candidate * as follows:
  - True peak location proposal for peak birth:
    $$s_{*}=g_{s_{*}}(U_{1};s_{j},s_{j+1})\in (s_{j},s_{j+1}),$$
    where g_s__^*^_ (*U*_1_; *s_j_*, *s_j_*_+1_) is a one-to-one mapping from random variable *U*_1_ to peak location s_*_ given *s_j_* and *s_j_*_+1_. We take g_s___*__ (*U*_1_; *s_j_*, *s_j_*_+1_) to be (*s_j_* + *s_j_*_+1_*g*_1_(*U*_1_))/(1 + *g*_1_(*U*_1_)), or (s_*_ − *s_j_*)/(*s_j_*_+1_ − s_*_) = *g*_1_(*U*_1_), where *g*_1_(·) is any monotonic function with domain [0, 1] and range [0, ∞), and *U*_1_ ~ *U* [0, 1]. It can be seen that, s_*_ is a monotonically increasing function of *U*_1_. We simply use *g*_1_(*u*) = *u*/(1 − *u*), thus s_*_ = *s_j_* + (*s_j_*_+1_ − *s_j_*) *U*_1_, a uniform random variable ∈ (*s_j_*, *s_j_*_+1_).
  - Peak sample location variance proposal for peak birth:
    $$\sigma_{*}^{2}=g_{\sigma_{*}^{2}}\left(U_{2};\sigma_{j}^{2},\sigma_{j+1}^{2}\right),$$
    where *g*_σ_*_^2^_(*U*_2_;σ*_j_*^2^, σ*_j+_*_1_^2^) is a one-to-one mapping from random variable *U*_2_ to peak sample location variance σ_*_^2^ given σ*_j_*^2^ and σ*_j_*_+1_^2^. We take *g*_σ_*_^2^_ (*U*_2_;σ*_j_*^2^, σ*_j+_*_1_^2^) to be (σ*_j_*^2^σ*_j_*_+1_^2^)^1/2^ *g*_2_(*U*_2_) or (σ_*_/σ*_j_*)/(σ*_j_*_+1_/σ_*_) = *g*_2_(*U*_2_), where *g*_2_(·) is any monotonic function with domain [0, 1] and range [0, ∞), and *U*_2_ ~ *U* [0, 1]. It can be seen that, σ_*_^2^ is a monotonically increasing function of *U*_2_. We simply use *g*_2_(*u*) = *u*/(1 − *u*), thus σ^2^_*_ = (σ^2^*_j_* σ^2^*_j_*_+1_)^1/2^ *U*_2_/(1 minus; *U*_2_).
  - Peak sample false negative and false positive rate proposal for peak birth:
    $$({fn}_{*},{fp}_{*})={\tilde{g}}_{{fn}_{*},{fp}_{*}}(U_{3},U_{4};{fn}_{j},{fp}_{j},{fn}_{j+1},{fp}_{j+1}),$$
    where g̃_fn___*_,_ _fp___*__ (*U*_3,_ *U*_4_; *fn_j_*, *fp_j_*, *fn_j_*_+1_, *fp_j_*_+1_), is a one-to-one mapping from (*U*_3_, *U*_4_) to (fn__*__, fp__*__) given (*fn_j_*, *fp_j_*) and (*fn_j_*_+1_, *fp_j_*_+1_). Specifically, for peak *, we use O_*_ to represent “false negative or positive” odds and R_*_ to represent “false negative vs. positive” ratio, i.e.
    (4) $$O_{*}=({fn}_{*}+{fp}_{*})/(1-{fn}_{*}-{fp}_{*})\;\text{and }R_{*}={fn}_{*}/{fp}_{*},$$
    the false negative and false positive rate proposal is realized in two sequential steps:
    1. O_*_ proposal:
      (5) $$O_{*}={\left(O_{j}O_{j+1}\right)}^{1/2}g_{3}(U_{3}),$$
      i.e. (O_*_/*O_j_*)^1/2^/(*O_j_*_+1_/O_*_)^1/2^ = *g*_3_(*U*_3_), where *g*_3_(·) is any monotonic function with domain [0, 1] and range [0, ∞), and *U*_3_ ~*U* [0, 1]. It can be seen that, O_*_ is a monotonically increasing function of *U*_3_, we simply use *g*_3_(*u*) = *u*/(1 − *u*).
    2. R_*_ proposal:
      (6) $$R_{*}={\left(R_{j}R_{j+1}\right)}^{1/2}g_{4}(U_{4}),$$
      i. e. (R_*_/*R_j_*)^1/2^/(*R_j_*_+1_/R_*_)^1/2^ = *g*_4_(*U*_4_), where *g*_4_(·) is any monotonic function with domain [0, 1] and range [0, ∞), and *U*_4_ ~ *U* [0, 1]. It can be seen that, R_*_ is a monotonically increasing function of *U*_4_, we simply use *g*_4_(*u*) = *u*/(1 − *u*).
  Note that the constraint 0 ≤ fn_*_ + fp_*_ ≤ 1 holds under this proposal. The g̃_fn___*_,_ _fp___*__ (*U*_3,_ *U*_4_; *fn_j_*, *fp_j_*, *fn_j_*_+1_, *fp_j_*_+1_) function is a combination of O_*_ proposal and R_*_ proposal in this case. fn_*_ and fp_*_ are jointly proposed to meet the constraint. The Jacobian of transforming (*fn_j_*, *fp_j_*, *fn_j_*_+1_, *fp_j_*_+1_, *u*_3_, *u*_4_) into ((*fn_j_*, *fp_j_*, fn_*_, fp_*_, *fn_j_*_+1_, *fp_j_*_+1_) is calculated by chain rule (see appendix).
  When the insertion of the peak birth candidate is before the first peak or after the last peak, there are no real double neighbors. In this case, we take the duplicates of peak birth candidate’s unique succeeding or preceding neighbor as two virtual neighbors for proposal implementation. In this move type, we realize the sequential uniform lift for fn_*_ + fp_*_ and subsequent conditional uniform lift for fn_*_ within fn_*_ + fp_*_. We claim that, the peak birth proposal by “+” move type, along with the peak death proposal by the following “−” move type, constructs a symmetric transition, i.e. equally probable events (Proposition 3). So the acceptance probability in the Metropolis-Hastings algorithm within Gibbs sampler is simply
  $$\text{min}\left\{1,\frac{f(K,\;{\tilde{S}}_{K},\;{\tilde{\sigma }}_{K},\;\tilde{f}n_{K},\;\tilde{f}p_{K}\;[\text{after peak death proposal}]|\;Y\text{)}}{f(K,\;{\tilde{S}}_{K},\;{\tilde{\sigma }}_{K},\;\tilde{f}n_{K},\;\tilde{f}p_{K}\;[\text{before peak death proposal}]|\;Y\text{)}}\right\}.$$
3. For the “−” move type, the parameter proposal process is: If *K_old_* = *K_min_*, we go to 1) since the lower threshold is reached; if *K_old_* > *K_min_*, we randomly sample one from current *K_old_* peaks with equal probability 1/*K_old_*, say index *, to delete. Then we simply abandon the associated s_*_, σ_*_^2^,fn_*_ and fp_*_ for likelihood reconstruction. The acceptance probability in the Metropolis-Hastings algorithm within Gibbs sampler is simply
  $$\text{min}\left\{1,\frac{f(K,\;{\tilde{S}}_{K},\;{\tilde{\sigma }}_{K},\;\tilde{f}n_{K},\tilde{f}p_{K}\;[\text{after peak death proposal}]|\;Y\text{)}}{f(K,\;{\tilde{S}}_{K},\;{\tilde{\sigma }}_{K},\;\tilde{f}n_{K},\;\tilde{f}p_{K}\;[\text{before peak death proposal}]|\;Y\text{)}}\right\}.$$
  The “+/−” move type is demonstrated in Figure 2 for only peak location model.
4. For the “*S*” move type, the parameter proposal process is: We randomly sample one of current peaks, say *, with equal probability 1/*K_old_*, and take the two neighboring peak intervals which peak *separates as a fused interval. A peak location is uniformly randomly drawn within this fused interval for a new candidate peak to replace s_*_. The other parameters associated with this peak location mutation is kept unchanged, or they could be changed as a set within the uniformity framework. This is also a symmetric transition (Proposition 2). So the acceptance probability in the Metropolis-Hastings algorithm within Gibbs sampler is simply
  $$\text{min}\left\{1,\frac{f(K,\;{\tilde{S}}_{K},\;{\tilde{\sigma }}_{K},\;\tilde{f}n_{K},\;\tilde{f}p_{K}\;[\text{after peak mutation proposal}]|\;Y\text{)}}{f(K,\;{\tilde{S}}_{K},\;{\tilde{\sigma }}_{K},\;\tilde{f}n_{K},\;\tilde{f}p_{K}\;[\text{before peak mutation proposal}]|\;Y\text{)}}\right\}.$$
5. For the “*H*” move type, the posterior distribution of each σ*_k_*^2^ is Inverse-Gamma (ν′, η′), where ν′ = ν + (∑*_yij_* _∈_ *_peak k_*1)/2. η′ = 1/(1/η +∑*_yij_* _∈_*_peak k_* (*y_ij_* − *s_k_*)^2^/2). The joint posterior distribution of each (*fn_k_*, *fn_k_*, 1 − *fn_k_* − *fp_k_*) is Dirichlet (α_1_ + *n_fn,k_*, α_2_ + *n_fp,k_*, $\alpha_{3}+n_{{\bar{fnp,\;}}_{k}}$). We sample the whole set of these parameters once.

#### Remark

This algorithm is a random scan (by move type probabilities) version of Gibbs sampler introduced by Gelfand and Smith (1990) with generalized parameter components:

1. peak (described by location, peak sample location variance, false negative and positive rates) birth or death changes the number of parameter sets by move type “+” or “−” in the aforementioned parameter sampling proposal;
2. peak location mutation does not change the number of parameter sets by move type “S” in the aforementioned parameter sampling proposal;
3. peak sample location variance or false negative/positive rate sampling does not change the number of parameter sets by move type “H” in the aforementioned parameter sampling proposal.

The associated propositions and the detailed description of RGPMCMC are given in the appendix. The following proposition justifies the correct convergence.

#### Proposition

The induced Markov chain is irreducible, aperiodic, and ergodic.

##### Interpretation

In view of independent uniform proposals for non-peak parameter set and diverse move types, given any arbitrarily small neighborhood of a current state there is a positive probability that the chain lies in that neighborhood after one sampling iteration, thus the aperiodicity is verified; the irreducibility is established since the chain can move from any state to any other state one step at a time.

## 3. Simulation Study

In this section, we study the RGPMCMC performance under diverse circumstances and make comparison with other approaches in the literature.

### 3.1. Prior sensitivity analysis for RGPMCMC

**Figure 3:**
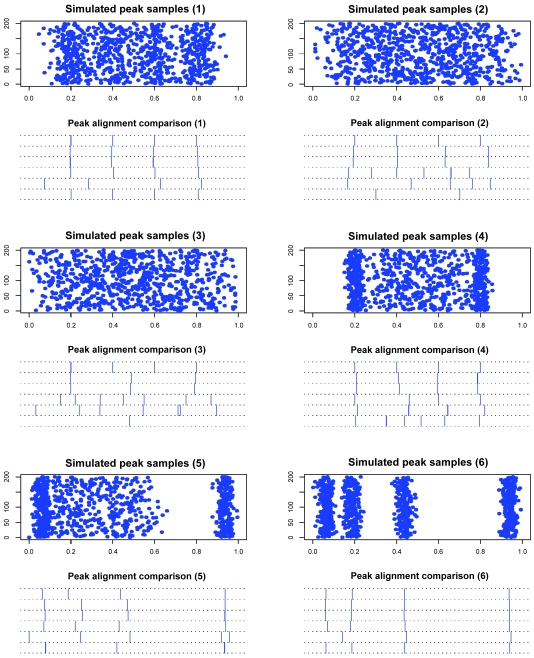
Simulations and Estimations (The simulation directly produces the peak samples for 200 patients which are plotted in each odd row of panels without the need for peak sample detection by data preprocessing, the y-axis is patient index and the x-axis is log(m/z). The alignments are compared by gridded walls given in each even row of panels. From top to bottom: true peaks, aligned peaks by RGPMCMC, aligned peaks by RJMCMC, aligned peaks by scale-space approach, aligned peaks by super-set approach and aligned peaks by PAM clustering algorithm.)

We consider different priors and study the discrepancy between the specified true peak locations and the estimated peak locations. Compaq Fortran 90 is our development package and we use IMSL Fortran Numerical Library to generate random numbers. 200 spectra are simulated for each of the six simulations, the set-ups and priors are listed in Table 1, where four true peaks are considered, the peak location vectors specify the true peak locations, the σ vectors specify the peak sample location variations at true peaks, the *fn* and *fp* vectors jointly specify the probabilities for us to simulate no peak sample (with probability *fn*), multiple peak samples (with probability *fp*), or single peak sample (with probability 1 − *fn* − *fp*) for each true peak from individual spectrum. The Inverse-Gamma and Dirichlet priors are for peak sample location variances and false negative and positive rates at putative true peaks. The peak number prior is *K* ~ *U* [1, 20], the starting peak number is 11, and move type probabilities are: π(+) = 0.45, π(−) = 0.45, π(*H*) = 0.05, and π(*S*) = 0.05. With burn-in 10,000 and thinning 1,000, each collection of 1,000 posterior samples takes several minutes on a PC powered by Celeron CPU. The results are given in Figure 3. In simulation 1, the true peaks are clearly clustered and the peak estimation is good. In simulation 2 with larger peak sample location variations and false positive/negative rates, the same number of peaks are identified as the true peak number under highly informative priors. By modifying peak sample location variation priors, we observe that, under large true variations, the peak estimation under less informative variation priors is worse than that under more informative variation priors. More uncertainties are introduced in simulation 3, and fewer peaks are identified than the true peak number. By modifying peak sample location variation priors, we observe that, when the variation priors are consistent with the true variation in terms of mean value, the peak number estimation seems to be better than inconsistent variation priors. Simulation 4 shows that, the informative peak sample location variation priors may lead to good estimation even when the peaks’ associated parameters are different, so does simulation 5, where unevenly distributed peaks are considered. In simulation 6, even when the parameter priors become much less informative, the peak estimation performs well, since the true peaks are sharply surrounded by corresponding peak samples. These observations show that, informative *a priori* knowledge is desirable for reliable estimation.

### 3.2. Comparison with some non-MCMC approaches

We first make comparisons with other non-MCMC algorithms represented by the recently developed scale-space approach (Yu et al. 2006a), super-set approach (Yu et al. 2006b), and Partitioning around Medoids (PAM) approach (Kaufman and Rousseeuw, 1990). For the six simulations in Section 3.1, the combined peak sample locations from 200 spectra along with the optimal cluster number minimizing the “median split silhouette” (Pollard and van der Laan, 2002) are taken as PAM inputs, S-plus function *pam* offers the medoid (cluster center) locations. These results are also included in Figure 3. Overall, RGPMCMC performs better than non-MCMC methods; the scale-space result is little better than the super-set result in terms of robustness; the PAM algorithm is not specifically designed for peak alignment, so it may perform poorly under certain circumstances, say simulations 2 and 3, where RGPMCMC can not recover all true peaks either.

### 3.3. Comparison with reversible jump Markov chain Monte Carlo

We apply the same simulated data in Section 3.1 and make comparison with reversible jump Markov chain Monte Carlo (RJMCMC) algorithm by Green (1995). RGPMCMC and RJMCMC differ in the method for proposing move type “+” and/or “−” (peak birth and/or death), where the former conditions on the active Markov chain by making use of equally probable peak birth and death proposals, while the latter makes use of additional variables to construct a one-to-one matching for dimension changing (details are given in the appendix). The same prior specifications for RGPMCMC in Section 3.1 are applied to RJMCMC. We consider two starting peak numbers, 11 and 1, both equally partitions the m/z range.

#### Initial peak number = 11

**Figure 4:**
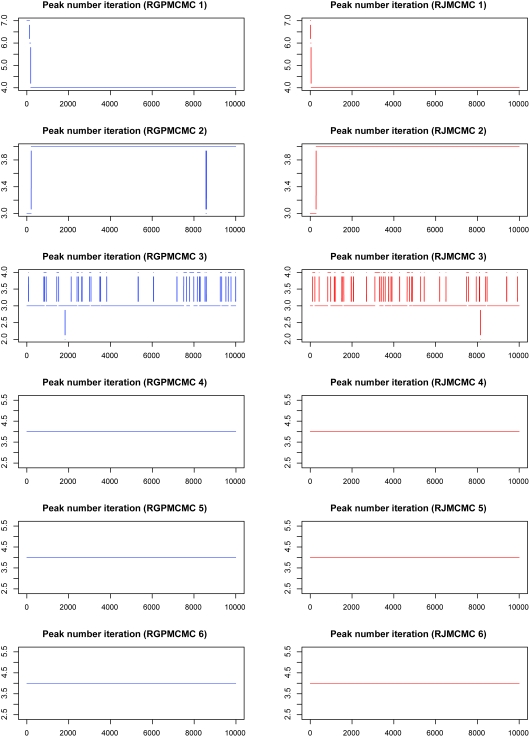
Peak Number Sampling Series by RGPMCMC and RJMCMC from Simulation Study (burn in = thinning = 1,000, the initial peak number = 11.)

**Figure 5:**
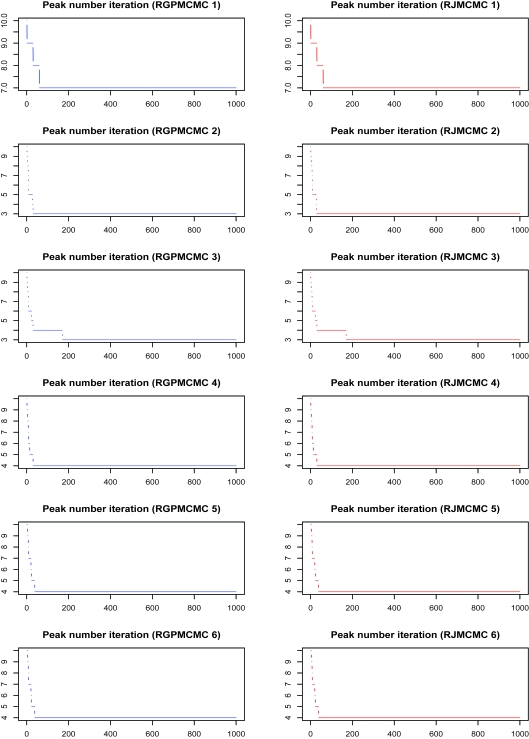
Peak Number Sampling Series by RGPMCMC and RJMCMC from Simulation Study (first 1,000 iterations with initial peak number 11)

1. Figure 3 also compares the alignments by RGPMCMC and RJMCMC, where no difference between RGPMCMC and RJMCMC exists.
2. The peak number iteration comparison is given in Figure 4, where the burn-in = thinning = 1,000. The third row of dual panels pinpoint 3 or 4 peaks, the 4:3 ratios are 0.124 and 0.122 for RGPMCMC and RJMCMC respectively, which is close to each other. The acceptance rate for peak birth and/or death proposals should be almost identical for these two algorithms.
3. The peak number iteration comparison before reaching reasonable peak number (1,000th iteration) can be seen from Figure 5. For these six simulations, peak birth and/or death, and peak mutation acceptance rates are compared in Table 2, which are close to each other.
4. The reasonable peak number is reached after almost the same number of iterations (~1,000) by GPMCMC and RJMCMC, which have similar efficiency for peak number identification.

#### Initial peak number = 1

**Figure 6:**
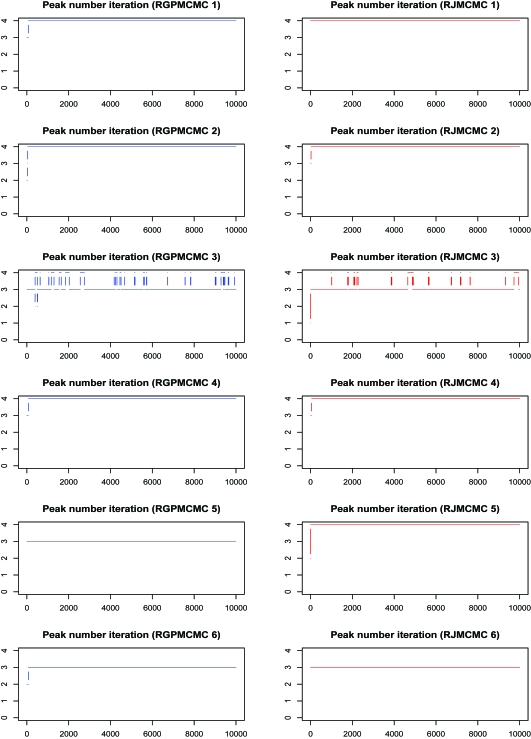
Peak Number Sampling Series by RGPMCMC and RJMCMC from Simulation Study (burn in = thining = 1,000 the initial peak number = 1.)

**Figure 7:**
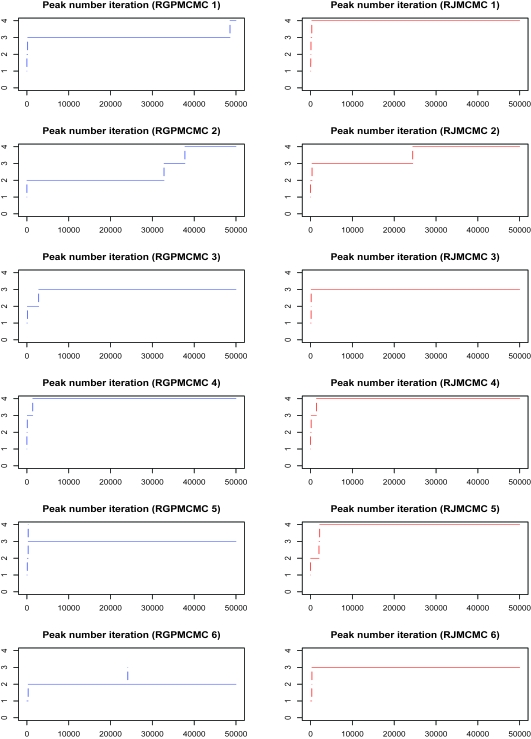
Peak Number Sampling Series by RGPMCMC and RJMCMC from Simulation Study (first 50,000 interations with initial peak number 1)

1. The peak number iteration comparison is given in Figure 6, where the burn-in = thinning = 1,000. The third row of dual panels pinpoint 3 or 4 peaks, the 4:3 ratios are 0.131 and 0.079 for RGPMCMC and RJMCMC respectively, where the ratio by RGPMCMC (0.131) is very close to peak number 11 case (0.124). Except for simulation 5, both RGPMCMC and RJM-CMC identify the same number of peaks. For simulation 6, they all identify 3 peaks other than the true 4 peaks.
2. The peak number iteration comparison before 50,000th iteration can be seen from Figure 7. For these six simulations, peak birth and/or death, and peak mutation acceptance rates are also compared in Table 2. The rates are still close to each other.

We find that, starting from a relatively large peak number is more capable of identifying true peaks by RGPMCMC and RJMCMC.

### 3.4. Mimic-MS simulation study

**Figure 8:**
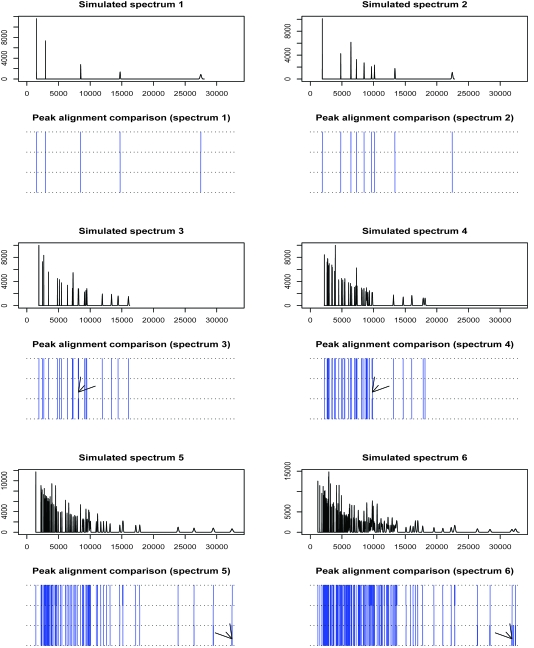
Mimic-MS Simulations and Estimations (The R simulator produces the spectrum profiles given in each odd row of panels, 100 random spectra were simulated for each of them for peak sample detection. The y-axis is intensity and the x-axis is m/z. After peak sample detection by data preprocessing, the alignments are compared by gridded walls given in each even row of panels. From top to bottom: true peaks, aligned peaks by RGPMCMC and aligned peaks by clustering algorithm.)

We mimic the mass spectra using the R package developed by Coombes et al. (2005b), where wide-ranging factors are considered to create the uncertainty, including the acquisition time resolution of the detector, the distribution of initial particle velocities, isotope distribution and others. Ideally, our six simulated mass spectrum groups have 5, 10, 20, 40, 80 and 160 peaks without incorporating any uncertainty and the R simulator produces six mean spectra, where the particles with large mass value (>20,000 Da) have broader hills due to more isotopes (Figure 8). Each mean spectrum leads to 100 uncertainty involved random replicative spectra subject to peak sample detection. For them we smooth spectrum with a predefined Gaussian function (window size of 15), search local maxima in the local neighborhood of 15 data points as peak samples, the minimal intensity value of peaks should be not smaller than 100. The RGPMCMC pinpoints 5, 9, 18, 35, 62 and 112 peaks (Inverse-Gamma (6,500) is peak sample location variance prior, Dirichlet (5,5,90) is false negative and positive rate prior, the starting peak numbers are 10, 20, 40, 80, 160 and 320); the clustering method in Tibshirani et al. (2004) pinpoints 5, 9, 19, 36, 69 and 174 peaks (the tuning parameters are selected as suggested in Tibshirani et al. (2004)). The alignment comparison is also given in Figure 8, where the clustering method tends to identify redundant peaks at large masses (the arrows in the bottom panels of Figure 8), while RGPMCMC performs better in this region; both methods identify less peaks in certain dense regions (bottom panels in Figure 8), while RGPMCMC sometimes combine too concentrated peaks into one peak (the arrows in the middle panels of Figure 8). Overall, these two approaches have similar performance in this simulation scenario.

## 4. Application to Real Data

**Figure 9:**
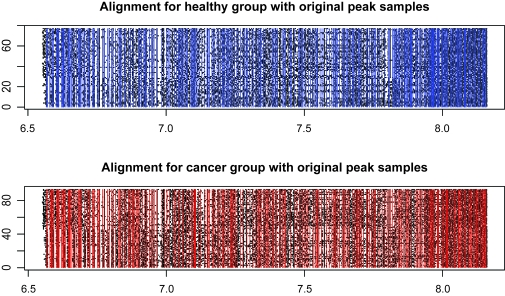
Aligned Peaks with Peak Sample Background (y-axis: patient index, x-axis: log(m/z). The vertical lines represent aligned peaks (biomarker profile) for each group, the dots in the background are the deteced peak samples for all patients by data preprocessing.)

**Figure 10:**
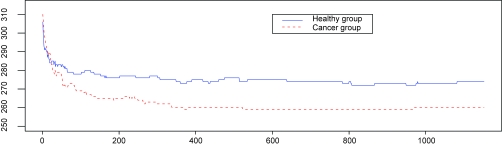
Sampling Series of Peak Number (y-axis: peak number, x-axis: iteration index. The healthy group seems to have more peaks than cancer group.)

**Figure 11:**
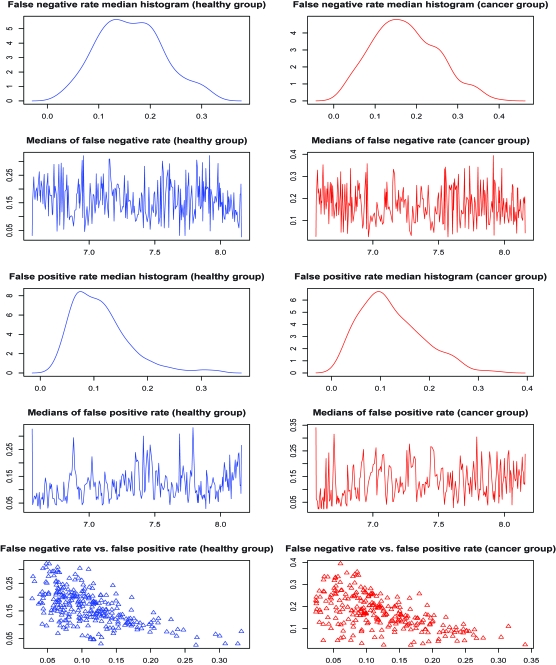
False Negative Rate and False Positive Rate Estimation (The left panel of first row is the histogram of false negatives at aligned peaks from healthy group, the right panel of first row is the histogram of false negatives at aligned peaks from cancer group; the left panel of second row shows the false negative medians at aligned peaks (represented by log (m/z)) from healthy group, the right panel of second row shows the false negative medians at aligned peaks (represented by log (m/z)) from cancer group; the next two rows are for false positives; the bottom row of panels show (false positive rate [x-axis], false negative rate [y-axis]) at aligned peaks for healthy group and cancer group.)

**Figure 12:**
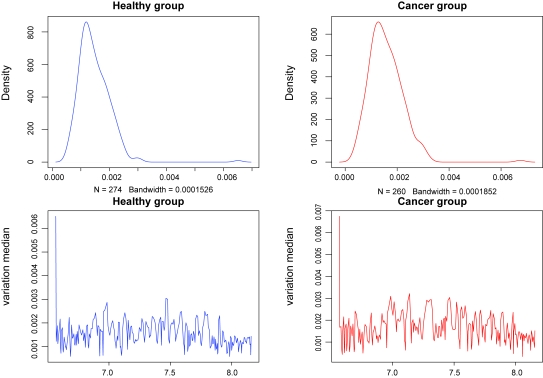
Posterior Medians of Peak Sample Location Variation (The upper left panel is the histogram of peak sample location variation medians from healthy group, the upper right panel is the histogram of peak sample location variation medians from cancer group; the lower left panel shows peak sample location variation medians (healthy group) at aligned peaks represented by log (m/z), the lower right panel shows peak sample location variation medians (cancer group) at aligned peaks represented by log(m/z).)

We model the same ovarian cancer data source as used by Wu et al. (2003) (available on-line at http://bioinformatics.med.yale.edu/MSDATA), where the healthy group has 77 patients and the cancer group has 93 patients. The individual spectrum has tens of thousands of (m/z, intensity) pairs and looks like a more complicated and error-involving version of those simulated profiles in Section 3.4 (Figure 8). The data preprocessing on original spectra involves baseline subtraction, smoothing, intensity normalization and peak sample detection by local maxima as described in Section 3.4. The median of the original peak sample numbers after pre-processing is 249 for the healthy group and 241 for the cancer group. The values of log(m/z) range from 6.565 to 8.200. We use move type probabilities (π(+) = 0.45, π(−) = 0.45, π(*H*) = 0.05, π(*S*) = 0.05) and the interval constraint *d* = 10^−5^. If this constraint is not met at one iteration, we simply resample the parameters. Richardson and Green (1997) observed that, proper posterior distributions may be not possible under fully noninformative priors, so we apply (α_1_ = 5, α_2_ = 5, α_3_ = 90) to Dirichlet priors, and (ν = 3, η = 2^6^) to Inverse-Gamma priors. Different peak number priors, either truncated Poisson [100, 600|λ] or Uniform [100, 600] lead to similar results. The starting peak number is *K* = (*K_min_* + *K_max_*)/2, the initial peak locations are equal *K*- partition of log(m/z) range, burn-in is 10,000, and thinning is 1,000. We recommend to start with a relatively large peak number. The sampler approaches the reasonable peak number very quickly and usually sticks around and mostly does effective single peak mutations once approaching the true peak number. Occasional peak number jump ups are highly efficient for joint peak number and location estimation. The alignment results are given in Figure 9. The sampling series of peak number are given in Figure 10, where we empirically identify the modes of the posterior peak number distribution as 274 (healthy group) and 260 (cancer group). The false negative rate and false positive rate estimations are given in Figure 11 with a significant negative correlation. The posterior peak sample location variation medians are given in Figure 12, where the inconsistency of the first point possibly arises from edge effect, so do false negative and positive rate median plots (Figure 11). The average peak distance (~(8.200–6.565)/290 = 0.0058) dominates the estimated peak sample location variation (~0.001). The sampler takes several thousand iterations to approach the reasonable peak number, thus an adaptive strategy with varying move type probabilities may be more efficient than the brute birth/death dominating proposal after that time point. The collection of the posterior samples in Figure 10 takes several days on a PC powered by Celeron CPU.

## 5. Sample Classification

**Figure 13:**
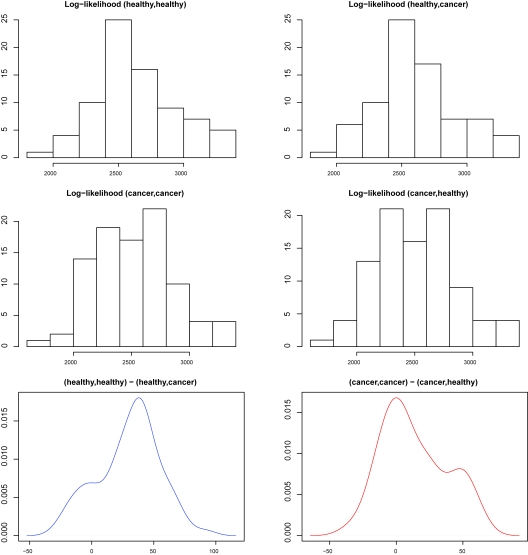
Hypothesis Test by Log-likelihood (The upper left panel is the log-likelihood histogram for healthy individuals with healthy group model, the upper right panel is the log-likelihood histogram for healthy individuals with cancer group model; the middle left panel is the log-likelihood histogram for cancer individuals with cancer group model, the middle right panel is the log-likelihood histogram for cancer individuals with healthy group model; the lower left panel is the log-likelihood difference histogram for “healthy individuals with healthy group model—healthy individuals with cancer group model”, the lower right panel is the log-likelihood difference histogram for “cancer individuals with cancer group model—cancer individuals with healthy group model”.)

Effective sample classification is as important as biomarker profile estimation. Wu et al. (2003) compared a number of sample classification methods without cross-validation and Tibshirani et al. (2004) reached error rates no less than 35%. Since the peak number is biologically variable between healthy and cancer groups, the current equal-peak-number based classifications may not be very suitable. Without considering cross-validation, we simply calculate the two log-likelihood functions for each preprocessed spectrum given fitted model (healthy group or cancer group) from Section 4, where the estimated parameters are taken from posterior medians. The histograms of log-likelihoods of all spectra from both healthy group and cancer group along with the log-likelihood difference histograms are given in Figure 13. From the log-likelihood difference empirical distributions in the bottom panels of Figure 13, we simply take the proportions of negative values as type I error rates: 28.6% for testing: Healthy vs. Cancer and 10.8% for testing: Cancer vs. Healthy, which are overly optimistic. Denote *ỹ* = (*y*_1_, *y*_2_, …, *y_N_*) as the spectrum peak sample location vector to be classified, *s̃_H_* = (*sh*_1_, *sh*_2_, …, *sh*_K*_H_*_) is the estimated true peak location vector for the healthy population, and *s̃_C_* = (*sc*_1_, *sc*_2_, …, *sc_K_*_*_c_*_ is the estimated true peak location vector for the cancer population (usually *K_H_* ≠ *K_C_*). We propose a minimum *L̄*_1_ distance sample classification rule. For each *sh_k_* (1 ≤ *k* ≤ *K_H_*), we identify its nearest neighbor *yn_hk_* from *ỹ* and form a *L*_1_ distance summand |*sh_k_* − *yn_hk_*|, the same rule applies to each *sc_k_* (1 ≤ *k* ≤ *K_C_*). To be conservative, we only use those deviations less than 0.003 (~half of the average peak interval) followed by arithmetic average over these selected estimated true peaks. The *L̄*_1_ distances between the new spectrum *ỹ* from healthy population and cancer population are

$${\bar{L}}_{1}(\tilde{y},H)=\frac{1}{K_{H}^{\prime }}\sum_{k=1}^{K_{H}}1_{selected}|sh_{k}-y_{n_{hk}}|$$

and

$${\bar{L}}_{1}(\tilde{y},C)=\frac{1}{K_{C}^{\prime }}\sum_{k=1}^{K_{C}}1_{selected}|sc_{k}-y_{n_{ck}}|,$$

where *K*′*_H_* is the number of selected true peaks from *s̃_H_* based on 0.003 threshold, and, *K*′*_C_* is the number of selected true peaks from *s̃_C_*, 1*_selected_* is the indicator function of selection. The class leading to smaller *L̄*_1_ would be predicted. Our 10 fold cross-validation is implemented as follows: we equally divide both the healthy and the cancer groups into ten disjoint pairs of testing set (*H_i_, C_i_*), (*i* = 1, …, 10). For each of these pairs, we combine the complementary nine sets as the corresponding healthy and cancer group training sets (*H*′*_i_*, *C*′*_i_*) (*i* = 1, …, 10), i.e. *H*′*_i_* = ∪_1 ≤_ *_k_* _≤ 10,_ *_k_*_≠_*_i_* *H_k_* and *C*′*_i_* = ∪_1 ≤_ *_k_* _≤ 10,_ *_k_* _≠_ *_i_C_k_*. The exact 10 fold cross-validation would be done for each of these testing pairs (*H_i_*, *C_i_*) from fitting (*H*′*_i_*, *C*′*_i_*), (*i* = 1, …, 10). The results are given in Table 3. The type I error rate for hypothesis test: *H*_0_: Healthy vs. *H*_1_: Cancer is around 38.97%, and the type I error rate for hypothesis test: *H*_0_: Cancer vs. *H*_1_: Healthy is around 27.96%, and the overall sample misclassification rate from 10 fold cross-validation is around 32.94%. Our strict 10-fold cross validation error rate is less than that of Tibshirani et al. (2004), which is actually not coming from a complete cross validation by observing that all spectra took part in the initial clustering. Comparing Table 3 and Figure 13, we conclude that, overlapping training and testing sets may favor individual spectrum with a bonus around 10% in terms of classification error rate. The conclusion drawn by Yasui et al. (2003) also holds here: an appreciable proportion of healthy samples may be incorrectly classified as cancer.

## 6. Discussion

In this article, we take a global viewpoint to avoid multiple edge effects under piecewise processing and incorporate flexible biomarker numbers to make our Bayesian model more accountable. The Jacobian term derivation, intensive death rate calculation, or lengthy recursive partition required by RJMCMC, CTMCMC and others in the literature may impede convenient application of Bayesian algorithm to change point identification (see the RJMCMC computational procedure in the appendix for example). For multiple change point identification problems where each segment has the same set of regulating parameters, we can see that, the superiority of RGPMCMC algorithm over other available algorithms is the most computational efficiency and simplicity by minor local adjustment of likelihood function and prior set armed with “naively informative” global treatment as introduced by the parameter sampling process in Section 2.2. Its competing computational performance has been demonstrated in this article by intensive comparison with others. Moreover, RGPMCMC can be easily modified to apply to multiple change point identification in circular domain (Liu et al. 2006) and others. Although our mass charge ratio (m/z) model already leads to promising sample classification, how to make use of relative intensity information beyond m/z value is a more challenging statistical problem, since peak samples in close proximity with disparate intensities are less likely to belong to the same putative true peak. Under the assumption of reproducibility and homogeneity of mass spectra, this algorithm is designed to be applied to each phenotype group separately (disease and control) at this point, leading to likely different peak location vectors for different phenotypes. Wu et al. (2006) observed that, a protein subset with considerable size, say 20~40 m/z features, may pose as signature between phenotypes, thus separate global statistical models are still desirable. Any peak sample detection protocol will cause inevitable peak sample location variation, false negative and false positive peak samples, which is obviously subject to statistical modeling. From algorithmic aspects, Green (1995) emphasized the importance of proposing parameter efficiently. Because the independent proposal from joint prior distribution of (*K*, θ*_K_*) is not very efficient, our proposal works on the joint infinitesimal space to achieve more efficiency by a more fair birth/death move. Although the RGPMCMC does not need intensive tuning parameter optimization, running MCMC properly is never a simple automatic task, since from simulation study we find that, highly informative prior specification consistent with the truth is desirable, for which a solution could be a local small scale study out of the whole spectra picture. The mass spectra’s quality and characteristics vary greatly depending on the platform, e.g. MALDI-TOF or SELDI-TOF, and certain experimental settings used for the measurements. This is not our concern here since it is not difficult to apply this global profile estimation algorithm to those spectra coming from the same source and enjoying high reproducibility and homogeneity. We anticipate that, the RGPMCMC developed in this article will shed light on a broad class of Bayesian multiple change point identification problems, not only MS data analysis. Lastly we emphasize that, diverse alignment problems arise from complicated scenarios in modern bioinformatics research. Beyond this m/z based mass spectra peak alignment which greatly benefits from Green (1995)’s seminal paper, Green and Mardia (2006) recently developed a novel Bayesian approach for simultaneous inference about the matching and the transformation between two protein 2D-gel images, and aligning active sites of proteins in three dimensions.

## Figures and Tables

**Table 1:** Simulation Configurations (IG: Inverse-Gamma prior for σ^2^, D: Dirichlet prior for (*fn*, *fp*, 1 − *fn* − *fp*)).

Peak location (*s*_1_, *s*_2_, *s*_3_, *s*_4_) = (1/5, 2/5, 3/5, 4/5											
(1)	σ	0.05	0.05	0.05	0.05	(2)	σ	0.10	0.10	0.10	0.10
	*fn*	0.10	0.10	0.10	0.10		*fn*	0.20	0.20	0.20	0.20
	*fp*	0.10	0.10	0.10	0.10		*fp*	0.20	0.20	0.20	0.20
		IG(5, 100), D(1, 1, 8)				IG(26, 4), D(40, 40, 120)	
(3)	σ	0.20	0.20	0.20	0.20	(4)	σ	0.02	0.08	0.08	0.02
	*fn*	0.30	0.30	0.30	0.30		*fn*	0.10	0.20	0.30	0.40
	*fp*	0.40	0.40	0.40	0.40		*fp*	0.10	0.20	0.30	0.40
		IG(6, 5), D(5, 5, 5)				IG(20, 6), D(5, 5, 10)	

**Peak location (*s*1,*s*2,*s*3,*s*4) = (1/16, 3/16, 7/16, 15/16)**											

(5)	σ	0.02	0.08	0.08	0.02	(6)	σ	0.02	0.02	0.02	0.02
	*fn*	0.10	0.20	0.30	0.40		*fn*	0.10	0.10	0.10	0.10
	*fp*	0.10	0.20	0.30	0.40		*fp*	0.10	0.10	0.10	0.10
		IG(20, 6), D(5, 5, 10)						IG(3, 2), D(2, 2, 2)	

**Table 2:** Acceptance Rate Comparison between RGPMCMC and RJMCMC.

Simulation	1	2	3	4	5	6
	First 1,000 iterations (the initial peak number = 11)					
peak birth (RGP)	0.00E-4	0.00E-4	0.00E-4	0.00E-4	0.00E-4	0.00E-4
peak birth (RJ)	0.00E-4	0.00E-4	0.00E-4	0.00E-4	0.00E-4	0.00E-4
peak death (RGP)	6.70E-3	1.55E-2	1.55E-2	1.33E-2	1.55E-2	1.55E-2
peak death (RJ)	6.70E-3	1.55E-2	1.55E-2	1.33E-2	1.55E-2	1.55E-2
peak mutation (RGP)	1.72E-2	3.53E-2	3.94E-2	1.30E-2	5.90E-3	2.73E-2
peak mutation (RJ)	1.93E-2	3.33E-2	4.42E-2	1.30E-2	5.90E-3	2.17E-2
	First 50,000 iterations (the initial peak number = 1)					
peak birth (RGP)	1.70E-4	1.26E-4	1.26E-4	1.70E-4	1.27E-4	8.50E-5
peak birth (RJ)	1.27E-4	1.26E-4	8.44E-5	1.28E-4	1.27E-4	8.43E-5
peak death (RGP)	0.00E-4	4.26E-5	2.12E-4	4.20E-5	2.53E-4	7.59E-4
peak death (RJ)	1.26E-4	4.25E-5	2.54E-4	4.20E-5	4.21E-5	7.21E-4
peak mutation (RGP)	1.71E-2	4.27E-2	4.08E-2	1.21E-2	8.96E-3	1.02E-2
peak mutation (RJ)	1.56E-2	3.04E-2	4.90E-2	8.51E-3	6.38E-3	1.41E-2

**Table 3:** Sample Classification Error Rates from 10 fold Cross-validation (The error rates represent the proportions of the healthy or cancer patients in the testing set which are misclassified.)

	Health group		Cancer group	
Cross-Validation	Identified peak number (Training set)	Error rate (Testing set)	Identified peak number (Training set)	Error rate (Testing set)
1	256	3/8	261	1/10
2	264	5/8	257	3/10
3	269	2/8	258	4/10
4	260	3/8	254	4/10
5	263	4/8	257	0/10
6	265	2/8	263	5/10
7	263	3/8	252	4/10
8	265	3/8	264	3/10
9	262	2/8	270	1/10
10	264	3/5	252	1/3

Overall		30/77		26/93

## Appendix: Proofs and Remark

### Proposition 1

*Consider the order statistics* (*U*_(1)_, …, *U*_(_*_n_*_)_*) of n uniformly distributed random variables U**_i_* *~ U[0, L], then conditionally U**_(k)_* *is uniformly distributed on [U**_(k_*_−_*_1)_**, U**_(k+1)_**]*.

#### Proof of Proposition 1

From $f_{U_{{\left(1\right)}_{,\ldots ,}}U_{\left(n\right)}}(u_{\left(1\right)}{,}_{\ldots },u_{\left(n\right)})=\frac{n!}{L^{n}}1_{\left(0<u(1)<\ldots <u(n)\right)}$. we get

(7) $$\begin{array}{l} f_{U_{\left(1\right)},\ldots ,U_{\left(i-1\right)},U_{\left(i+1\right)},\ldots ,U_{\left(n\right)}}(u_{\left(1\right)},\ldots ,u_{\left(i-1\right)},u_{\left(i-1\right)},\ldots ,u_{\left(n\right)})\\ =\int f_{U_{\left(1\right)},\ldots ,U_{\left(n\right)}}(u_{\left(1\right)},\ldots ,u_{\left(n\right)})du_{\left(i\right)}\\ =\int_{u_{\left(i-1\right)}}^{u_{\left(i+1\right)}}f_{U_{\left(1\right)},\ldots ,U_{\left(n\right)}}(u_{\left(1\right)},\ldots ,u_{\left(n\right)})du_{\left(i\right)} \end{array}$$

Thus, $f(u_{i}|u_{\left(-i\right)})=\frac{1}{u_{\left(i+1\right)}-u_{\left(i-1\right)}}$. This proposition is a statistical prototype for only peak birth location proposal.

### Proposition 2

Random single peak location mutation within its interval is a symmetric transition kernel.

#### Proof of Proposition 2

Assume two non-zero measured sets *ds* and *ds*′ are located within the interval of length *L*, we have

$$P(ds\to {ds}^{\prime })=\int_{ds}P(s\to {ds}^{\prime })=\frac{{ds}^{\prime }}{L}\frac{ds}{L}=\frac{ds}{L}\frac{{ds}^{\prime }}{L}=\int_{{ds}^{\prime }}P(s^{\prime }\to ds)=P({ds}^{\prime }\to ds).$$

### Random Grafting-pruning Markov Chain Monte Carlo (RGPMCMC) Proposition 3

Peak birth and death proposals introduced in “+” and “−” move types could be taken as a symmetric transition, and Jacobian terms is not needed by RGPMCMC for these two move types.

#### Illustration of Proposition 3

We look at the simple case for only peak location birth. Under Proposition 1,

1. For the move type “+”, if *K*_old_ < *K*_max_, then $\text{Pr}(K_{\text{old}}\to K_{\text{old}}+1,s_{K_{\text{old}}+1}\in A)=\frac{1}{K_{{\text{old}}^{+1}}}\times \frac{A}{L};\text{Pr}(s_{K_{\text{old}}+1}\in A,K_{\text{old}}+1\to K_{\text{old}})=\frac{A}{L}\times \frac{1}{K_{{\text{old}}^{+1}}}$. $\frac{A}{L}$ comes from probability measure integration of the new state for peak birth proposal.
2. For the move type “−”, if *K*_old_ > *K*_min_, then $\text{Pr}(s_{K_{\text{old}}}\in A,K_{\text{old}}\to K_{\text{old}}-1)=\frac{A}{L}\times \frac{1}{K_{\text{old}}};\text{Pr}(K_{\text{old}}-1\to K_{\text{old}},s_{K_{\text{old}}}\in A)=\frac{1}{K_{\text{old}}}\times \frac{A}{L}$; where *L* is the length of the selected peak interval and *A* is a Borel set within it. $\frac{A}{L}$ comes from probability measure integration of the old state for peak death proposal.

The peak birth/death proposal prescribed by the parameter sampling process in Section 2.2 takes account of current peak density information (Figure 2), e.g. dense peaks attract more attention. Green (1995) proposed the change point number and locations separately with birth/death proposal ratio *K*_old_+ 1, thus peak death gets more favorable as the peak number grows. When all true peaks are concentrated at one end, the peak birth proposal efficiency by a random draw within the whole domain may be low in view of the lack of peaks on the majority part of peak location domain. To illustrate that, Jacobian terms are unnecessary in RGPMCMC, and peak birth and death proposal could be taken as equally probable, or symmetric proposals, we map the individual parameter set (*s*_*_, σ_*_^2^, *fn*_*_, *fp*_*_) back onto the generating spaces of variables *U*_1_, *U*_2_ and (*U*_3_, *U*_4_). See “+” move type. The probability is only considered in the latter spaces. The product probability of certain measurable balls around the observed parameter set

(8) $$B(s_{*})\times B(\sigma_{*}^{2})\times B({fn}_{*},{fp}_{*}),$$

is corresponding to

(9) $$B^{\prime }(U_{1})\times B^{\prime }(U_{2})\times B^{\prime }(U_{3},U_{4}),$$

where the *B*′(·)s are the mapped generating sets for observed parameter sets *B*(·)s. For peak birth, 1/(*K**_old_* + 1) is the probability of randomly selecting a peak interval * out of current *K**_old_* + 1 ones, on which a new candidate peak grows up with a certain shape (Borel set (8)) specified by associated parameter set (*s*_*_, σ_*_^2^, *fn*_*_, *fp*_*_). Hereafter we work on two parts of interested parameters: peak interval index * (I) and describing set (*s*_*_, σ_*_^2^, *fn*_*_, *fp*_*_) (D). “+” move type realized “I” and “D” parts sequentially. If we try to propose the reverse process (peak death) by tracing the presumed peak birth proposal: a current “I”, say *, is selected with probability 1/*K**_old_* (*K**_old_* equals the preceding *K**_old_* + 1 below (9) in peak birth case) with “D”, say (*s*_*_,σ_*_^2^, *fn*_*_, *fp*_*_). The presumed birth proposal for this peak is to be replayed blindly and independently. Any non-exact overlap with the original peak birth parameters represents an ineffective peak death proposal/no proposal and only an exact overlap represents an effective peak death proposal, with the same probability as random peak birth. The ineffective peak death proposal/no proposal leads to proposal-freezing Markov chain, which is useless for Metropolis-Hastings algorithm based inference. In this sense, we claim that the proposed peak birth/death move type is a symmetric transition without the need for Jacobian terms since we physically work on the generating parameter spaces with identical dimensions. In other words, conditioning on choosing one candidate peak interval for birth proposal, we imagine a target is randomly hit somewhere on the local *E* equal-partition within this interval, with landing (active peak birth proposal, the proposal takes effect) probability $\frac{1}{E}$, each shoot accounts for one active proposal; conditioning on choosing one candidate peak for death proposal, we imagine a random shoot on this fused peak interval holding the peak (see “*S*” move type in the aforementioned parameter sampling process, Section 2.2) with hit (active peak death proposal, the proposal takes effect) probability $\frac{1}{E}$, where *E* equal-partition is done within this fused interval. The peak birth/death proposal is statistically equivalent to: take no action with probability $\pi (-)\frac{E-1}{E}$, or propose a random peak birth or death with probability $\pi (+)\frac{1}{E}$ or $\pi (-)\frac{1}{E}$. The proposal in Section 2.2 discards unnecessary proposal freezing during Markov chain inference. Step 2) and step 3) realize one type of symmetric transitions (equally probable random landing and hit); step 5) realizes another type of symmetric transitions (equally probable landings within an interval). Random grafting-pruning Markov chain Monte Carlo (RGPMCMC) comes from the birth/death proposal which acts like randomly grafting or pruning a plant: node (peak for MS data) birth/death proposal is applied to the plant stem, the set of sub-branches, i.e. the describing parameters (s_*_, σ_*_^2^, *fn*_*_ and *fp*_*_), are lifted independently from the randomly selected stem interval; the random hit probability is identical to birth probability by imagining independent random shootings at each sub-branch within the same generating space for sub-branch birth. The peak birth and death proposals should be applied alternatively in a probabilistic manner. If we happen to choose to randomly delete one branch, then we may randomly add this very branch in the same place in the preceding birth proposal; on the other hand, if we happen to choose to randomly add one branch, then we may randomly delete this very branch in the succeeding death proposal, the equally probable branch birth/death is realized physically. The balance could also be justified by MS data analysis: among clearly clustered peaks, it is equally difficult to add another peak into any blank interval, or to delete any peak already well established. The grafting step 2) is quite flexible by requiring randomly harvesting a branch from the garden, which could be done by designing any convenient one-to-one mapping peak birth proposal function as discussed by the parameter sampling process in Section 2.2. Compared with CTMCMC, we simplifies the acceptance rate as only a local adjustment, e.g. for peak birth we only reassign the local peak samples to three true peaks (one is peak birth in between and the other two are its neighbors) plus an additional prior set for this new peak with all others unchanged, and vice versa for peak death. Adaptive move type probabilities may decrease possible high autocorrelation after the reasonable peak number is approached. The informative Dirichlet prior is to leash the number of putative peaks. Possible hyperparameters in hierarchical Bayesian analysis may induce another move type. To sum up, RGPMCMC has mathematical rigor and intuitive justification. The split/combine move by Richardson and Green (1997) may be designed to satisfy symmetric transition in terms of triple-integration, while Cappé, Robert and Rydén (2003) illustrated that, the difference between birth/death move and split/combine move is minor.

### Reversible Jump Markov chain Monte Carlo (RJMCMC) Formulation in MS Peak Alignment

For peak birth proposal, we randomly select a location within m/z domain, the subsequent σ_*_^2^, *O*_*_ and *R*_*_ proposal follows the parameter sampling process prescribed in Section 2.2, the acceptance probability of peak birth is

min{1,(likelihood ratio)×(prior ratio)×(proposal ratio)×(Jacobian)}.

The likelihood ratio is calculated from (2). The prior ratio becomes

(10) $$\begin{array}{l} \frac{p(K+1)}{p(K)}\frac{2(K+1)(2K+3)}{{\left({\left(m/z\right)}_{max}-{\left(m/z\right)}_{min}\right)}^{2}}\frac{\left(s_{*}-s_{j})(s_{j+1}-s_{*}\right)}{s_{j+1}-s_{j}}\frac{1}{\Gamma (\nu )\eta^{\nu }}{\left(\sigma^{2}\right)}^{-(\nu +1)}\text{exp}\{-1/(\sigma^{2}\eta )\}\\ \times \frac{\Gamma (\alpha_{1}+\alpha_{2}+\alpha_{3})}{\Gamma (\alpha_{1})\Gamma (\alpha_{2})\Gamma (\alpha_{3})}{fn}_{*}^{\alpha_{1}}{fp}_{*}^{\alpha_{2}}{\left(1-{fn}_{*}-{fp}_{*}\right)}^{\alpha_{3}}; \end{array}$$

the proposal ratio is

$$\frac{d_{K+1}({\left(m/z\right)}_{max}-{\left(m/z\right)}_{min})}{b_{K}(K+1)},$$

note that we have *b**_K_*=*d**_K_*_+1_. For peak sample location variance proposal: (σ*_j_*^2^, σ*_j_*_+1_^2^, *u*_1_) → (σ*_j_*^2^, σ_*_^2^, σ*_j_*_+1_^2^) the Jacobian is

$$\frac{{\left(\sigma_{*}^{2}+{\left(\sigma_{j}^{2}\sigma_{j+1}^{2}\right)}^{1/2}\right)}^{2}}{\sigma_{j}\sigma_{j+1}}$$

We now derive the Jacobian for proposing new false negative rate *fn*_*_ and false positive rate *fp*_*_ from (5) and (6). By notation (4), we have

(11) $${fn}_{*}=\frac{O_{*}}{\left(1+O_{*})(1+R_{*}\right)}\text{and }{fp}_{*}=\frac{O_{*}R_{*}}{\left(1+O_{*})(1+R_{*}\right)},$$

where both *O*_*_ and *R*_*_ are functions of (*fn**_j_*, *fp**_j_*, *fn**_j_*_+1_, *fp**_j_*_+1_, *u*_3_, *u*_4_). For transformation

(12) $$({fn}_{j},{fp}_{j},{fn}_{j+1},{fp}_{j+1},u_{3},u_{4})\to ({fn}_{j},{fp}_{j},{fn}_{*},{fp}_{*},{fn}_{j+1},{fp}_{j+1}),$$

we only need work on ∂*fn*_*_/∂(*fn**_j_*, *fp**_j_*, *fn**_j_*_+1_, *fp**_j_*_+1_, *u*_3_, *u*_4_) and ∂*fp*_*_/∂(*fn**_j_*, *fp**_j_*, *fn**_j_*_+1_, *fp**_j_*_+1_, *u*_3_, *u*_4_) by chain rules, since others are simple identities. For example,

(13) $$\frac{\partial {fp}_{*}}{\partial {fn}_{j}}=\frac{\partial {fp}_{*}}{\partial O_{*}}\frac{\partial O_{*}}{\partial {fn}_{j}}+\frac{\partial {fp}_{*}}{\partial R_{*}}\frac{\partial R_{*}}{\partial {fn}_{j}}.$$

Omitting tedious algebraic derivation, we get the following partial derivatives

(14) $$\begin{array}{l} \frac{\partial {fn}_{*}}{\partial {fn}_{j}}=\frac{1}{2}\frac{R_{*}}{1+R_{*}}\frac{1}{{\left(1+O_{*}\right)}^{2}}{\left(\frac{O_{j+1}}{O_{j}}\right)}^{1/2}\frac{u_{3}}{1-u_{3}}\frac{1}{{\left(1-({fn}_{j}+{fp}_{j})\right)}^{2}}+\frac{1}{2}\frac{O_{*}}{1+O_{*}}\frac{1}{{\left(1+R_{*}\right)}^{2}}{\left(\frac{R_{j+1}}{R_{j}}\right)}^{1/2}\frac{u_{4}}{1-u_{4}}\frac{1}{{fp}_{j}}\\ \frac{\partial {fn}_{*}}{\partial {fp}_{j}}=\frac{1}{2}\frac{R_{*}}{1+R_{*}}\frac{1}{{\left(1+O_{*}\right)}^{2}}{\left(\frac{O_{j+1}}{O_{j}}\right)}^{1/2}\frac{u_{3}}{1-u_{3}}\frac{1}{{\left(1-({fn}_{j}+{fp}_{j})\right)}^{2}}-\frac{1}{2}\frac{O_{*}}{1+O_{*}}\frac{1}{{\left(1+R_{*}\right)}^{2}}{\left(\frac{R_{j+1}}{R_{j}}\right)}^{1/2}\frac{u_{4}}{1-u_{4}}\frac{{fn}_{j}}{{\left({fp}_{j}\right)}^{2}}\\ \frac{\partial {fn}_{*}}{\partial {fn}_{j+1}}=\frac{1}{2}\frac{R_{*}}{1+R_{*}}\frac{1}{{\left(1+O_{*}\right)}^{2}}{\left(\frac{O_{j}}{O_{j+1}}\right)}^{1/2}\frac{u_{3}}{1-u_{3}}\frac{1}{{\left(1-({fn}_{j+1}+{fp}_{j+1})\right)}^{2}}+\frac{1}{2}\frac{O_{*}}{1+O_{*}}\frac{1}{{\left(1+R_{*}\right)}^{2}}{\left(\frac{R_{j}}{R_{j+1}}\right)}^{1/2}\frac{u_{4}}{1-u_{4}}\frac{1}{{fp}_{j+1}}\\ \frac{\partial {fn}_{*}}{\partial {fp}_{j+1}}=\frac{1}{2}\frac{R_{*}}{1+R_{*}}\frac{1}{{\left(1+O_{*}\right)}^{2}}{\left(\frac{O_{j}}{O_{j+1}}\right)}^{1/2}\frac{u_{3}}{1-u_{3}}\frac{1}{{\left(1-({fn}_{j+1}+{fp}_{j+1})\right)}^{2}}-\frac{1}{2}\frac{O_{*}}{1+O_{*}}\frac{1}{{\left(1+R_{*}\right)}^{2}}{\left(\frac{R_{j}}{R_{j+1}}\right)}^{1/2}\frac{u_{4}}{1-u_{4}}\frac{{fn}_{j+1}}{{\left({fp}_{j+1}\right)}^{2}}\\ \frac{\partial {fn}_{*}}{\partial u_{3}}=\frac{R_{*}}{1+R_{*}}\frac{1}{{\left(1+O_{*}\right)}^{2}}{\left(O_{j}O_{j+1}\right)}^{1/2}\frac{1}{{\left(1-u_{3}\right)}^{2}}\\ \frac{\partial {fn}_{*}}{\partial u_{4}}=\frac{O_{*}}{1+O_{*}}\frac{1}{{\left(1+R_{*}\right)}^{2}}{\left(R_{j}R_{j+1}\right)}^{1/2}\frac{1}{{\left(1-u_{4}\right)}^{2}} \end{array}$$

and

(15) $$\begin{array}{l} \frac{\partial {fp}_{*}}{\partial {fn}_{j}}=\frac{1}{2}\frac{1}{1+R_{*}}\frac{1}{{\left(1+O_{*}\right)}^{2}}{\left(\frac{O_{j+1}}{O_{j}}\right)}^{1/2}\frac{u_{3}}{1-u_{3}}\frac{1}{{\left(1-({fn}_{j}+{fp}_{j})\right)}^{2}}-\frac{1}{2}\frac{O_{*}}{1+O_{*}}\frac{1}{{\left(1+R_{*}\right)}^{2}}{\left(\frac{R_{j+1}}{R_{j}}\right)}^{1/2}\frac{u_{4}}{1-u_{4}}\frac{1}{{fp}_{j}}\\ \frac{\partial {fp}_{*}}{\partial {fp}_{j}}=\frac{1}{2}\frac{1}{1+R_{*}}\frac{1}{{\left(1+O_{*}\right)}^{2}}{\left(\frac{O_{j+1}}{O_{j}}\right)}^{1/2}\frac{u_{3}}{1-u_{3}}\frac{1}{{\left(1-({fn}_{j}+{fp}_{j})\right)}^{2}}+\frac{1}{2}\frac{O_{*}}{1+O_{*}}\frac{1}{{\left(1+R_{*}\right)}^{2}}{\left(\frac{R_{j+1}}{R_{j}}\right)}^{1/2}\frac{u_{4}}{1-u_{4}}\frac{{fn}_{j}}{{\left({fp}_{j}\right)}^{2}}\\ \frac{\partial {fp}_{*}}{\partial {fn}_{j+1}}=\frac{1}{2}\frac{1}{1+R_{*}}\frac{1}{{\left(1+O_{*}\right)}^{2}}{\left(\frac{O_{j}}{O_{j+1}}\right)}^{1/2}\frac{u_{3}}{1-u_{3}}\frac{1}{{\left(1-({fn}_{j+1}+{fp}_{j+1})\right)}^{2}}-\frac{1}{2}\frac{O_{*}}{1+O_{*}}\frac{1}{{\left(1+R_{*}\right)}^{2}}{\left(\frac{R_{j}}{R_{j+1}}\right)}^{1/2}\frac{u_{4}}{1-u_{4}}\frac{1}{{fp}_{j+1}}\\ \frac{\partial {fp}_{*}}{\partial {fp}_{j+1}}=\frac{1}{2}\frac{1}{1+R_{*}}\frac{1}{{\left(1+O_{*}\right)}^{2}}{\left(\frac{O_{j}}{O_{j+1}}\right)}^{1/2}\frac{u_{3}}{1-u_{3}}\frac{1}{{\left(1-({fn}_{j+1}+{fp}_{j+1})\right)}^{2}}+\frac{1}{2}\frac{O_{*}}{1+O_{*}}\frac{1}{{\left(1+R_{*}\right)}^{2}}{\left(\frac{R_{j}}{R_{j+1}}\right)}^{1/2}\frac{u_{4}}{1-u_{4}}\frac{{fn}_{j+1}}{{\left({fp}_{j+1}\right)}^{2}}\\ \frac{\partial {fp}_{*}}{\partial u_{3}}=\frac{1}{1+R_{*}}\frac{1}{{\left(1+O_{*}\right)}^{2}}{\left(O_{j}O_{j+1}\right)}^{1/2}\frac{1}{{\left(1-u_{3}\right)}^{2}}\\ \frac{\partial {fp}_{*}}{\partial u_{4}}=\frac{O_{*}}{1+O_{*}}\frac{1}{{\left(1+R_{*}\right)}^{2}}{\left(R_{j}R_{j+1}\right)}^{1/2}\frac{1}{{\left(1-u_{4}\right)}^{2}}. \end{array}$$

Where, *O**_j_* =(*fn**_j_* + *fp**_j_*)/(1 − *fn**_j_* − *fp**_j_*), *R**_j_* = *fn**_j_*/*fp**_j_*. After simplifications, we get the Jacobian

(16) $$\frac{O_{*}{\left(O_{*}+{\left(O_{j}O_{j+1}\right)}^{1/2}\right)}^{2}{\left(R_{*}+{\left(R_{j}R_{j+1}\right)}^{1/2}\right)}^{2}}{{\left(O_{j}O_{j+1}\right)}^{1/2}{\left(R_{j}R_{j+1}\right)}^{1/2}{\left(1+R_{*}\right)}^{2}{\left(1+O_{*}\right)}^{3}}.$$
